# Total mastectomy and chest reconstruction for a rapidly progressing giant phyllodes tumor with skin necrosis: a case report

**DOI:** 10.1186/s40792-015-0082-9

**Published:** 2015-09-14

**Authors:** Aya Banno, Akihiko Shimada, Kenichiro Aga, Hiroki Harada, Takuji Kaburagi, Hiroaki Seki, Nobutaka Yasui, Hidetoshi Matsumoto

**Affiliations:** Department of Surgery, Keiyu Hospital, 3-7-3 Minatomirai Nishiku, Yokohama, 220-8521 Japan

**Keywords:** Phyllodes tumor, Skin necrosis, Mastectomy

## Abstract

Phyllodes tumors are rare fibroepithelial neoplasms of the breast. In the literature, borderline or malignant tumors have been reported to present with unusual characteristics including a short clinical history and extremely rapid tumor growth. Skin necrosis and infection sometimes accompanies these malignancies. Giant phyllodes tumors have a good prognosis when treated with total mastectomy, but reconstruction of the chest wall has been a challenge because of the need for a wide-range excision.

We report a case of a malignant phyllodes tumor that was initially diagnosed as borderline because sudden growth of the tumor contrarily induced sparse to moderate stroma cellularity in the sections of the tumor that were biopsied. Total mastectomy without axillary lymph node resection and chest wall reconstruction using a full-thickness mesh skin graft was performed. The patient has remained free from infection and recurrence for over a year since diagnosis.

## Background

Phyllodes tumors are relatively rare fibroepithelial neoplasms most prevalent in women in their 40s and 50s [1]. The histology consists of three types: benign, malignant, and the least prevalent, borderline. The average size of these tumors is 4 cm, but 20 % are called giant phyllodes tumors because of their abnormal diameter, growing to more than 10 cm [2]. In the literature, borderline or malignant tumors have been reported to present with unusual characteristics, including a short clinical history and aggressive growth [3–5]. Skin necrosis and infection sometimes accompanies these malignancies. Giant phyllodes tumors have a good prognosis after total mastectomy, but reconstruction of the chest wall has been a challenge, as they require wide-range excision.

We report a case of a malignant phyllodes tumor that was initially diagnosed as borderline because the rapid growth contrarily caused sparse to moderate cellularity in portions of the tumor, including the biopsied specimen.

## Case presentation

A 47-year-old Japanese hospital employee presented to our surgery clinic with the chief complaint of a giant mass in her right breast. Her medical history was remarkable only for asthma; her last attack had been more than 20 years ago. The results of the last checkup for breast cancer, 7 years ago, had been negative. She first felt a mass of the size of her thumbnail 5 years prior to the visit. Four years prior to admission, she incidentally palpated the tumor, and noted slight growth, after the family dog collided with her breast, but she did not seek medical consultation because her breasts were symmetrical in size.

One month before her visit, the size of the right breast started to increase rapidly. She experienced fatigue, dizziness, and heaviness in the right breast because of the weight of the tumor. One week previously, the skin covering the mass became necrotic and had a foul odor. Her condition deteriorated, and family members finally insisted that she consult a clinician.

**Fig. 1:**
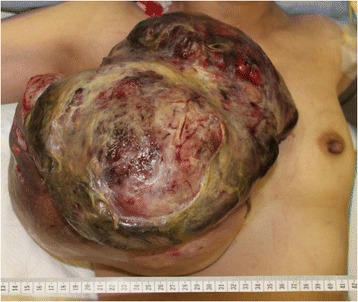
Gross examination findings before surgery. The mass was approximately 30 cm long

On arrival, her vital signs were as follows: blood pressure, 104/60 mm Hg; heart rate, 115 beats per minute; axillary temperature, 38.3 °C; and respiratory rate, 28 breaths per minute. Physical examination revealed a 30-cm, multifocal, sphere-shaped mass in the right breast. Findings of the gross examination before treatment are shown in Fig. 1. Owing to anemia, malnutrition, and the weight of the mass, she could not maintain a supine position or walk more than short distances.

Most of the skin of the right breast was necrotic and had a foul odor. Culture of the exudate was strongly positive for *Pseudomonas aeruginosa*. Laboratory findings identified a prominently abnormal complete blood count: white blood cell of 15,300/μl, hemoglobin level of 6.3 g/dl, and platelet count of 536,000/μl. Malnutrition and hyponatremia were obvious from the blood biochemistry tests. She was immediately admitted for diagnosis and treatment.

**Fig. 2:**
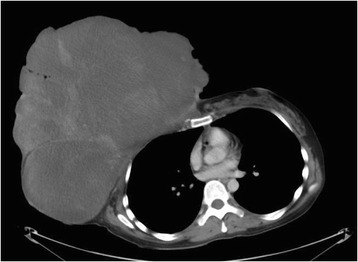
Contrast-enhanced computed tomography findings. The tumor measured 20 × 28 × 28 cm and was segmented. Free air is visible. The boundary between the tumor and the pectoralis major muscle is unclear. The axillary lymph nodes were mildly enlarged, with a maximum diameter of 10 mm on the right side. No distant metastases are noted

Contrast-enhanced computed tomography (Fig. 2) and magnetic resonance imaging showed a hemorrhagic giant mass with a well-defined border. The pectoralis major muscle was atrophic, but there was no sign of chest wall invasion or distant metastasis. A diagnosis of phyllodes tumor of borderline malignancy was made on the basis of the core needle biopsy findings.

**Fig. 3:**
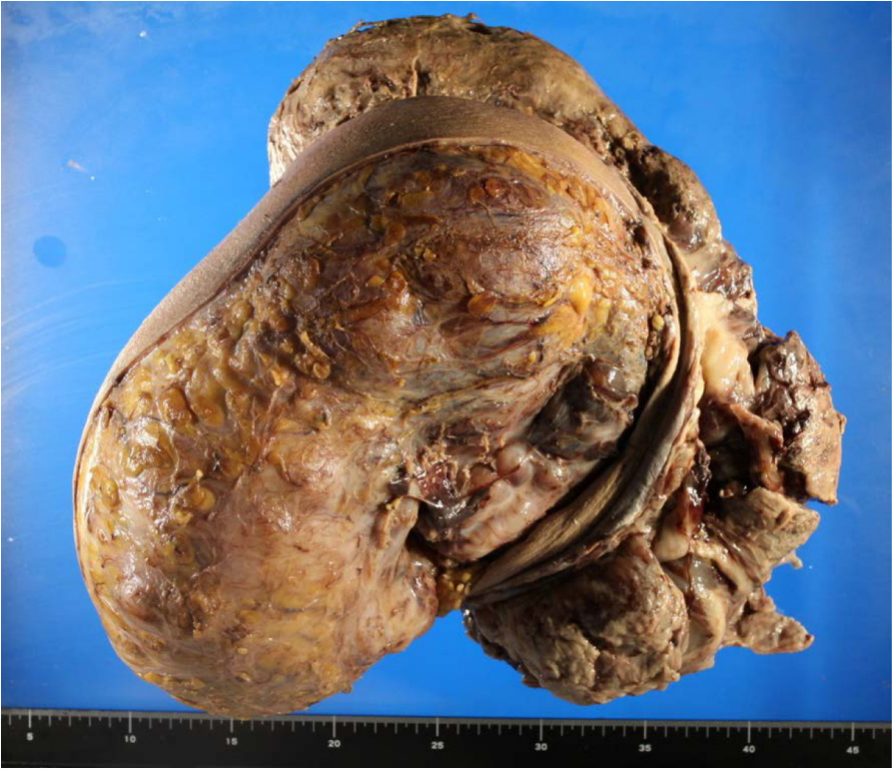
Gross appearance of the cut surface of the transected tumor. The tumor measured 35 × 34 cm and weighed 7640 g

On the fourth day of admission, we performed total excision of the right breast. The pectoralis major muscle was significantly thin and atrophic, but macroscopic features of invasion were not observed. After an elaborate cleaning of the excision site, we placed a full-thickness mesh skin graft, made by puckering the tissue (dog ear) after suturing the skin to the chest wall. We completed the procedure by protecting the wound with sterilized gauze. The enucleated lesion weighed 7640 g. The gross appearance of the cut surface is shown in Fig. 3.

For infection control, we thoroughly washed the necrotic skin over the tumor every day with 4–5 l of tap water until the day of operation. After resecting the tumor, we elaborately cleaned the site with 5 l of sterile saline. Antibiotics were administered during the perioperative period.

**Fig. 4:**
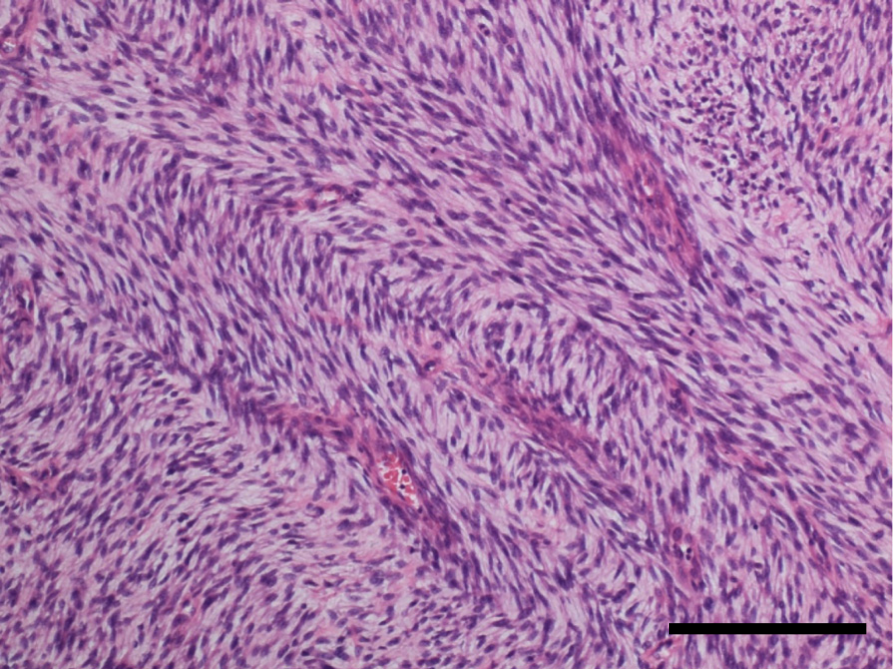
Histopathological findings. The photomicrograph shows long, spindle cells forming a herringbone pattern in most hypercellular areas (hematoxylin and eosin staining). The bar is 200 μm long

Pathological findings revealed a 35 × 34-cm tumor. Microscopically, most of the mass was composed of hypercellular stroma in leaf-like structures; a herringbone pattern was observed in the densest areas (Fig. 4). On immunostaining, the tumor was negative for estrogen and progesterone receptors as well as for desmin and S-100. It was diffusely positive for CD-10 in the hypercellular regions. Only a few cells were positive for actin and CD34. Reactivity for MIB-1 was observed in 30 % of the cells, and a few cells reacted with a p53 antibody. Cutaneous invasion was not observed, but the chest wall skeletal muscle was invaded in limited regions. The final diagnosis was a malignant phyllodes tumor; however, the stroma cellularity was variable and only a few sections presented with moderate stroma density.

**Fig. 5:**
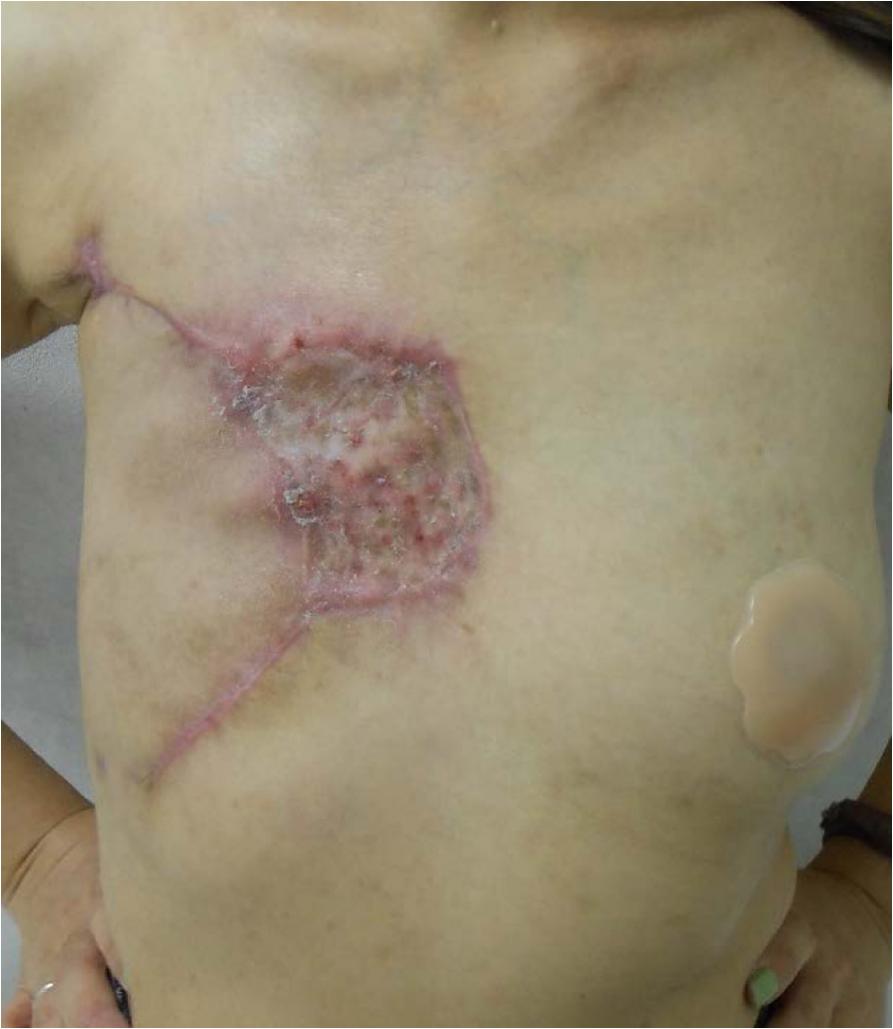
Findings at the 2-month follow-up. The full-thickness skin graft can be seen firmly attached to the chest wall

No critical complications occurred during hospitalization. The patient was discharged on the 11^th^ day after surgery. Despite widespread infection of the site, we confirmed firm attachment of the skin graft to the chest wall 2 months after discharge. Recurrence has not been observed for more than 1 year after the initial presentation (Fig. 5).

### Discussion

Phyllodes tumors are relatively rare neoplasms with an estimated incidence of 2.1 per 1 million women. They constitute 0.3–0.9 % of all breast tumors and 2–3 % of fibroepithelial lesions [1, 6]. Women between 35 and 55 years of age are the most commonly affected. Some studies suggest that the prevalence is higher in Latin American, Caucasian, and Asian populations [1, 2].

Phyllodes tumors are classified into three types according to the criteria of Azzopardi and Salvadori: benign, borderline, and malignant. These three histotypes are seen in approximately 35–60 %, 10–15 %, and 25–35 % of cases, respectively [2, 7, 8].

The patient’s typical complaints are a painless, well-circumscribed, mobile mass with an average diameter of 4–5 cm [2]. Many studies have reported tumors with an unexpected behavior such as growth or necrosis, despite the low rate of malignancy. In some patients, a small lesion may be present for several years, but it becomes clinically apparent only after a sudden increase in size [3–5]. These giant tumors can grow to more than 10 cm in size, and they account for 20 % of all phyllodes tumors. Most are malignant or borderline on pathological examination [2]. Skin necrosis is often observed in malignant cases and rarely in borderline neoplasms, although cutaneous invasion was not examined in previous studies [3–5].

These pathological findings were in accordance with our case; therefore, rapid skin extension is suspected to be attributable to cutaneous necrosis in giant phyllodes tumors. Chest wall invasion is also an uncommon finding, for which extended excision of the pectoralis muscle is recommended if the fascia or the muscle is infiltrated [2, 8].

Rapid growth misled our initial diagnosis of this case because some sections of the tumor were not dense enough to be diagnosed as a malignant phyllodes tumor. We assume that these portions were extended by the speedy expansion caused by the malignancy that composed most of the tumor.

Previous studies have proven an association of p53 and MIB-1 in malignant phyllodes tumors, although a correlation between the extent of protein expression and the size of the tumor or the speed of growth has not been clearly indicated [9, 10].

Mastectomy is the first choice of treatment for giant phyllodes tumors. Lymph node metastasis is detected in less than 1 % of cases; therefore, routine axillary clearance is not recommended in any of the histotypes, including the malignant type [2]. As for the pectoralis major muscle, this case may be at risk for recurrence in the chest wall because focal invasion was observed in the final pathology report. Nonetheless, we did not perform further resection of the pectoralis major muscle because the invasion was limited and the operation site is not fully covered with skin. We believe the mesh skin graft will be helpful in early detection in case of recurrence. We plan to regularly examine the patient in the follow-up clinic.

The Marlex mesh and the latissimus dorsi muscular/myocutaneous flap are the two most commonly recommended methods for reconstruction of the chest wall [4, 2]. However, both can have major complications. The Marlex mesh can cause severe post-operative infection, and its use is contraindicated for infected sites, such as in the reported case. Latissimus dorsi muscular flaps are invasive and costly treatments and can delay recovery of the patient.

## Conclusions

We report a malignant phyllodes tumor that was first diagnosed as a borderline tumor that presented with rapid growth and skin necrosis within an extremely short period of time (1 month). The patient underwent mastectomy of the right breast. Full-thickness skin grafts may be an uncomplicated and safe method for reconstruction of the damaged chest wall.

## Consent

The patient provided informed consent for the publication of this report and any accompanying images.
